# Validation of the English and Chinese versions of the Quick-FLIC quality of life questionnaire

**DOI:** 10.1038/sj.bjc.6602399

**Published:** 2005-02-08

**Authors:** Y-B Cheung, K-S Khoo, J Thumboo, G-Y Ng, J Wee, C Goh

**Affiliations:** 1 Division of Clinical Trials and Epidemiological Sciences, National Cancer Centre, Singapore; 2 Department of Medical Oncology, National Cancer Centre, Singapore; 3 Department of Rheumatology and Immunology, Singapore General Hospital, Singapore; 4 Department of Palliative Medicine, National Cancer Centre, 11 Hospital Drive, Singapore 169610

**Keywords:** quality of life, shortening, validation

## Abstract

A useful measure of quality of life should be easy and quick to complete. Recently, we reported the development and validation of a shortened Chinese version of the Functional Living Index – Cancer (FLIC), which we called the Quick-FLIC. In the present study of 327 English-speaking and 221 Chinese-speaking cancer patients, we validated the English version of the Quick-FLIC and further assessed the Chinese version. The 11 Quick-FLIC items were administered alongside the 11 remaining items of the full FLIC, but there appeared to be little context effect. Validity of the English version of the Quick-FLIC was attested by its strong correlation with two other measures of quality of life, and its ability to detect differences between patients with different performance status and treatment status (each *P*<0.001). Its internal consistency (alpha=0.86) and test–retest reliability (intraclass correlation=0.76) were also satisfactory. The measure was responsive to changes in performance status (*P*<0.001). The Chinese version showed similar characteristics. The Quick-FLIC behaved in ways that are highly comparable with the FLIC, even though the Quick-FLIC comprised only 11 items whereas the FLIC comprised 22. Further research is required to see whether the use of shorter instruments can improve data quality and response rates, but the fact that shorter instruments place less burden on the patients is itself inherently important.

A clinically useful measure of health-related quality of life (HRQoL) should be easy and quick to complete (Higginson and Carr, 2001). Lengthy questionnaires impose a heavy burden on patients, who may already be feeling unwell because of their illnesses. Furthermore, longer questionnaires may give a lower response rate and a higher incidence of missing values than shorter ones (Dorman *et al*, 1997; Axelsson and Sjoden, 1999; Edwards *et al*, 2004). Efforts have been made by various research teams to abridge long questionnaires. A noted example is the shortening of the SF-36 to the SF-12 (Ware *et al*, 1996).

Recently, we reported the development and validation of an abbreviated Chinese version of the Functional Living Index – Cancer (FLIC) and termed the resultant questionnaire the Quick-FLIC (Cheung *et al*, 2004a). Drawing on data from a sample of Chinese-speaking patients who filled out the Chinese version of FLIC, and guided by factor analysis results, we selected a limited subset of items to form the Quick-FLIC in Chinese (Cheung *et al*, 2003a). This was then validated using an independent sample (Cheung *et al*, 2004a). Although the FLIC was developed to assess the overall level of HRQoL, factor analysis showed that its 22 items reflected five domains of well-being: physical, psychological, social, family and symptoms (Ruckdeschel and Piantadosi, 1994; Cheung *et al*, 2003a). The Quick-FLIC has kept this structure. Its 11 items include three on physical well-being (‘feel well’, ‘pain or discomfort’ and ‘feel uncomfortable’), and two on each of the other domains: psychological (‘discouraged’ and ‘frightened of the future’), social (‘willing to see friends’ and ‘willing to see those closest to you’), family (‘hardship on those closest to you’ and ‘satisfaction with work/housework’) and symptoms (‘nausea’ and ‘pain related to illness’). Although the Quick-FLIC consisted of only 11 items, its measurement properties were very similar to the original 22-item Chinese version of FLIC. This Chinese version was derived from the English original of FLIC, which was translated into Chinese and adapted for use in Singapore (Goh *et al*, 1996; Cheung *et al*, 2003b). This study examines whether it is possible to use the same 11 items of the English original to assess HRQoL in English, and looks at validity, reliability, internal consistency, and sensitivity to change of the English version of the Quick-FLIC.

The English and Chinese languages are two of the most widely used languages in the world (Graddol, 2004). Patients in many cancer centres in Europe and North America have a variety of ethnic backgrounds and primary languages (Tchen *et al*, 2003). In Singapore, a multiethnic society mainly consisting of ethnic Chinese, Malay and Indian, about 71% of the residents are literate in English and 65% in Chinese, and about one-third of the population is bilingual in both (Singapore Department of Statistics, 2000). The availability of the Quick-FLIC in both languages will facilitate research in multiethnic societies as well as multicentre studies and cross-cultural research.

The data set was derived from a study of about 1300 cancer patients in Singapore who spoke either English or Chinese to compare the characteristics of three major quality of life questionnaires, namely the FLIC, Functional Assessment of Cancer Therapy – General (FACT-G) and the European Organisation for Research and Treatment of Cancer Core Quality of Life Questionnaire (EORTC QLQ-C30). Although the primary purpose of this study was not related to the Quick-FLIC, we capitalised on this data set to study the English version of Quick-FLIC. One challenge may be that the validity and reliability of these 11 items administered as part of the FLIC could have been affected by the other FLIC items. However, recent studies suggested little context effect in quality of life assessment (Kemmler *et al*, 1999; Cheung *et al*, 2004b). Furthermore, we also used the same 11 items in Chinese to re-evaluate the Chinese version of Quick-FLIC. The results of this Chinese Quick-FLIC as administered in the context of the 22-item FLIC were compared with those of the Chinese Quick-FLIC administered as an independent instrument (Cheung *et al*, 2004a). Obtaining similar results would testify to the lack of a context effect in the present study.

## MATERIALS AND METHODS

### Design

The National Cancer Centre, Singapore provides care to approximately 70% of the cancer patients seen in the public health-care institutions in Singapore. The study was approved by the Ethics Committee of the Centre. An incomplete block design was used (Senn, 1993), in which participants were randomised to receive one of the following three questionnaire packages: (1) FLIC and FACT-G, (2) FLIC and EORTC QLQ-C30 or (3) FACT-G and EORTC QLQ-C30. We chose not to use a complete block design of having each patient answer all three questionnaires because our experience suggested that some patients might not be able or willing to spend so much time and concentration on this task. Owing to logistic considerations, the randomisation used days rather than individuals as units. At 4 weeks after the baseline interview, the same questionnaire package was sent to each patient, together with a prepaid return envelope. The present analysis only included patients who self-administered questionnaire packages 1 or 2, which included the FLIC. The design for data collection was determined by the primary purpose of comparing the measurement properties of the FLIC, EORTC QLQ-C30 and FACT-G, details of which will be reported in another manuscript.

### Patient recruitment

Patients were recruited from the Centre from September 2003 to May 2004. They were approached while they were in the waiting areas of the specialist outpatient clinics, the ambulatory treatment unit and the therapeutic radiology department. The inclusion criteria were: literate in either English or Chinese, aged 18 years or older, and willing to give written informed consent. The participants were heterogeneous in their characteristics, for example, having different types of cancer. This is suitable for the present purpose as the FLIC and Quick-FLIC are designed for application to all cancer patients. The participants could choose to answer either an English or a Chinese questionnaire according to their preference. Participants were requested to self-administer the questionnaires. Upon request by the patients, interviews would be administered by one of the two research coordinators of the project. Only ethnic Chinese patients were included in the present analysis so that the English and Chinese Quick-FLIC data were not confounded by ethnicity.

### Instruments

The FACT-G version 4 and the EORTC QLQ-C30 version 3 were used. The FLIC has been modified in two aspects for use in Singapore (Goh *et al*, 1996; Cheung *et al*, 2003b). Firstly, the word ‘cancer’ was replaced by the word ‘illness’ in the questionnaire because some patients, especially older ones, might not know their diagnosis and their families might not want them to be aware of it. Neither the FACT-G nor the EORTC QLQ-C30 mention the word cancer. Secondly, the visual analogue scale (VAS) was replaced by a seven-point Likert format scale because the VAS was difficult for some patients to complete, especially the older and less educated ones. Similar modifications of the FLIC have also been reported in other countries (Takeda and Uki, 1994; Conner-Spady *et al*, 2001). Each questionnaire package began with a page on questions about demographic and health characteristics, including ECOG performance score (Blagden *et al*, 2003) and whether the patients were receiving chemotherapy and/or radiotherapy.

### Statistical analysis

Individual items were recoded in a way such that a higher score meant better quality of life. Missing values in the FACT-G, FLIC and EORTC QLQ-C30 were imputed by the half-rule (Cella, 1997). For ease of comparison, we transformed scores for each scale used in this study into a 0–100 scale rubric (Fairclough, 2002). Owing to the small number of patients with an ECOG score equal to 4, in the analysis scores 3 and 4 were combined as one category. Treatment status was classified as whether the patient was currently on chemotherapy and/or radiotherapy or not. Tumour sites with prevalence smaller than 5% in this sample were combined and labelled as ‘others’.

Data from the English and Chinese questionnaires were analysed separately. Concurrent validity was examined by Pearson's correlation coefficient (*r*) between the Quick-FLIC score, the FACT-G total score, the EORTC QLQ-C30 global score and the 22-item version FLIC score. Previous studies have established a significant correlation between the FACT-G, EORTC QLQ-C30 and the FLIC (e.g., Cella *et al*, 1993). Since we hypothesised that the Quick-FLIC behaved in ways similar to the FLIC, we expected the Quick-FLIC to have a significant correlation with the FACT-G and EORTC QLQ-C30. Analysis of variance (ANOVA) was used to compare differences in mean Quick-FLIC values between groups of participants with different ECOG performance status and treatment status. A significant difference in mean values (*P*<0.05) was considered evidence for known-groups validity. Education is often found to be a powerful predictor of general health and psychological well-being (Cox *et al*, 1993; Cheung, 2002). If the Quick-FLIC specifically measures the impact of cancer and cancer treatment, it should not be strongly associated with education. ANOVA was used for comparison of education level. Reliability in terms of Cronbach's alpha was estimated. Test–retest reliability was estimated by intraclass correlation (ICC), limiting to participants who at the follow-up assessment reported the same ECOG status as they did in the baseline interview. We assessed the changes in the Quick-FLIC scores in relation to the change in ECOG performance status by ANOVA. The FLIC was analysed in the same way to provide a comparison with the Quick-FLIC for the purpose of assessing whether the proposed instrument could use fewer items to obtain the same results.

As our recent article showed (Cheung *et al*, 2004a), a sample size of 190 was required to satisfy various aspects of validating an instrument. The sample size here for each language was larger because the primary purpose of comparing the three instruments required it.

## RESULTS

### Recruitment results

A total of 2198 patients were approached, among whom 1317 (60%) consented to participate, including 1180 ethnic Chinese subjects. Out of the 1180, 778 (66%) were entered into the packages that contained the FLIC: 395 filled out the package containing the FLIC and EORTC and 383 the FLIC and FACT-G. A total of 195 questionnaires were administered by interviewers and 20 by proxies; the latter were excluded from the present analysis. One subject was excluded due to missing covariate values and 14 due to missing FLIC and Quick-FLIC scores. The total number of subjects included in this analysis was 548, of whom 285 filled out the package containing the FLIC and EORTC QLQ-C30 and 263 the FLIC and FACT-G. The numbers of patients who chose to answer the English and Chinese questionnaires were 327 and 221, respectively.

### Descriptive summary

Table 1 shows the demographic and clinical characteristics of the patients who filled out the English and Chinese questionnaires. Table 2 gives the mean and standard deviation (s.d.) values of the HRQoL measures. The mean and s.d. of the Quick-FLIC scores were almost identical to those of the FLIC in the English sample. These two means and s.d. values were also similar to the Chinese sample. There were fewer observations of FACT-G total and EORTC QLQ-C30 global scores because of the incomplete block design.

### Validity

The correlation coefficient (95% CI), *r*, between the Quick-FLIC and FLIC scores was 0.97 (0.96–0.98) in the English sample and also 0.97 (0.96–0.98) in the Chinese sample. In the English sample, the correlation coefficient between the Quick-FLIC and FACT-G was 0.73 (0.64–0.79); that between the Quick-FLIC and EORTC QLQ-C30 was 0.77 (0.70–0.83). The corresponding values in the Chinese sample were 0.81 (0.72–0.87) and 0.71 (0.61–0.79), respectively.

**Figure 1:**
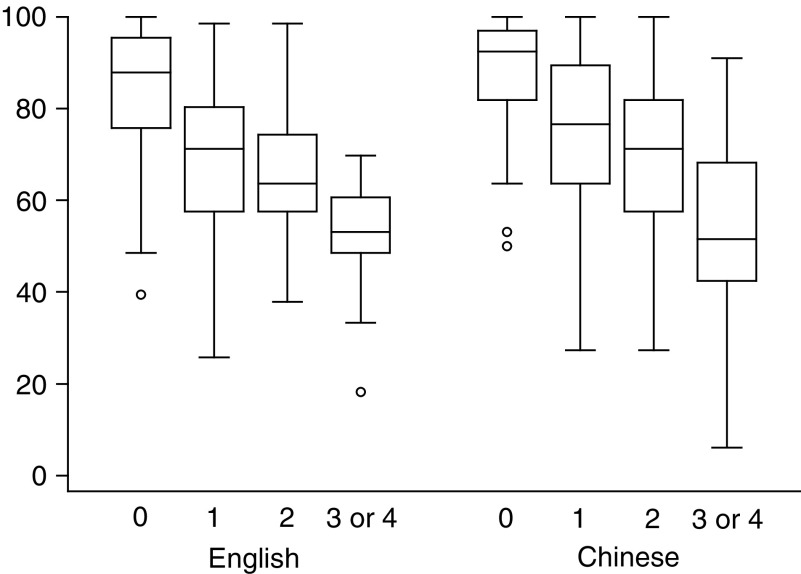
Box–Whisker plot of Quick-FLIC scores (*y*-axis) by ECOG performance status (*x*-axis) and versions of questionnaire. Boxes represent interquartile ranges; whiskers represent maximum and minimum values (or adjacent values as defined by Tukey, 1977).

Figure 1 presents the distribution of Quick-FLIC scores by ECOG performance status. In both the English and Chinese samples, a clear gradient of Quick-FLIC scores in relation to performance status was seen. Linear regression found that for each step of decline in performance status, the mean Quick-FLIC value declined by 11.0 points (95% CI −13.0 to −9.0) in the English sample and 10.6 points (−12.8 to −8.3) in the Chinese sample (each *P*<0.001). The FLIC behaved similarly: for each step of decline in performance status, the mean FLIC value declined by 10.7 points (95% CI −12.6 to −8.9) in the English sample and 11.1 points (−13.1 to −9.0) in the Chinese sample (each *P*<0.001).

Table 3 shows the mean HRQoL scores by treatment status and educational background. Patients with different treatment status showed significantly different mean Quick-FLIC scores (*P*<0.001). People with different educational background showed similar Quick-FLIC scores (*P*=0.520). In each stratum of treatment or education status, the Quick-FLIC and FLIC showed similar mean values; the ANOVA results were also comparable. In the Chinese sample, approximately the same results were obtained.

### Sensitivity to change

Table 4 shows mean Quick-FLIC values in relation to changes in ECOG performance status. Of the 548 patients, 388 (71%) responded to the follow-up survey. In both the English and Chinese-speaking samples, changes in mean Quick-FLIC scores were significantly associated with changes in performance status categories in the expected direction (each *P*<0.001). Furthermore, the magnitude of change in Quick-FLIC and FLIC scores was similar in each language version.

### Reliability

Using baseline data, Cronbach's alpha of the Quick-FLIC was 0.86 and 0.87 in the English and Chinese-speaking samples, respectively (Table 5). Those of the FLIC were slightly higher. Test–retest reliability was 0.76 and 0.82 in the two samples. The FLIC had very similar test–retest reliability values.

## DISCUSSION

The shortening of measures of self-reported outcome has been practised quite widely in educational and psychological research but surprisingly, has not been common in studies of quality of life of cancer patients (Coste *et al*, 1997). Research in other therapeutic areas has demonstrated the practicability of reducing the number of questions in composite measurement scales by 60–70% (Ware *et al*, 1996; Moran *et al*, 2001). Recently, we extracted 11 items of the Chinese version of the FLIC to form a Quick-FLIC questionnaire in Chinese and we validated this new questionnaire in an independent sample of Chinese-speaking patients in Singapore (Cheung *et al*, 2003a, 2004a). It follows logically to evaluate whether the same 11 items of the FLIC in English could form an English version of Quick-FLIC. We utilised a study in which patients chose to answer the English and Chinese versions of the FLIC to perform this analysis.

As we did not administer the Quick-FLIC questionnaire with 11 items only in English, there is a potential that the results might be affected by the context effect provided by the 22-item FLIC. However, recent studies suggested that the context of HRQoL assessment has little effect on HRQoL scores (Kemmler *et al*, 1999; Cheung *et al*, 2004b). Furthermore, a comparison of the findings of the Chinese Quick-FLIC in this study with our previous study, where the 11 items Quick-FLIC was administered as an independent instrument showed similar results. For instance, the correlation coefficient between the Chinese Quick-FLIC and FACT-G here was 0.81, whereas in the previous study it was 0.78 (Cheung *et al*, 2004a). This shows that the context did not have a big influence.

We have demonstrated that the English version of the Quick-FLIC correlated strongly with the FACT-G total and EORTC QLQ-C30 global scores, giving support to its concurrent validity. The Quick-FLIC scores behaved in the expected manner in relation to ECOG performance status and treatment status, testifying to its known-groups validity. It has been argued that if a cancer HRQoL measure truly measures something specific to cancers, it should not be correlated with education level (Cheung *et al*, 2004a). In this sample, we found no relation between the Quick-FLIC and education level, thus supporting its divergent validity. The longitudinal data attested to the responsiveness to changes in performance status and test–retest reliability of the Quick-FLIC. Streiner and Norman (1989) suggested that a composite measurement scale should have a test–retest reliability of 0.75 or above. The Quick-FLIC passed this standard and had a strong Cronbach's alpha as well.

Results from the Chinese questionnaires were similar to those from the English questionnaires. The FLIC and Quick-FLIC had very similar measurement properties, suggesting that little information was lost in shortening the 22-item FLIC to the 11-item Quick-FLIC. The only exception was that the FLIC had a higher Cronbach's alpha value, both in the English and the Chinese-speaking samples. However, it is known that this statistic is dependent not only on the strength of association among the items but also on the number of items in the scale (Streiner and Norman, 1989). Hence, the higher alpha values of FLIC do not indicate inferiority on the part of the Quick-FLIC.

The Quick-FLIC was derived from the version of the FLIC validated for use in Singapore, where the word ‘cancer’ was changed to the word ‘illness’. This was done because a high proportion of cancer patients have not been told that they have cancer and families do not want them to know, for example, 41% of patients referred to a hospice service did not know their diagnosis (Tay *et al*, 1994). Inclusion of the word ‘cancer’ in HRQoL questionnaires does not seem to be a requirement among oncology researchers, as the EORTC QLQ-C30 and the FACT-G both do not involve the word ‘cancer’. As the above results have shown, the Quick-FLIC has the desired measurement properties in this group of cancer patients even though the word ‘cancer’ was not used in the questionnaire. It may be interesting to further examine the Quick-FLIC, either with or without the word ‘cancer’, in other oncology populations to see whether the findings can be replicated.

We validated the Quick-FLIC in a heterogeneous sample of cancer patients because this and the other instruments involved are developed for application to all cancer patients rather than some clinical or demographic subgroups. Our choice of sample matched the intended usage of the instrument. Performance status is a very powerful determinant of HRQoL in cancer patients; other disease- or treatment-related factors tend to have limited effect (e.g. Aaronson *et al*, 1993). Our sample covered patients with a wide range of ECOG performance status, although numbers with ECOG score 4 was small. The results should be very generalisable except for very ill patients, who typically are excluded in the assessment of self-reported outcomes.

The English Quick-FLIC can be used to assess the quality of life of English-speaking cancer patients. As English and Chinese are two of the most widely used languages in the world (Graddol, 2004), the availability of both English and Chinese versions of the Quick-FLIC will expand its potential, especially for research in multiethnic societies and in multicentre studies.

Despite many advances in quality of life research in the last two decades, some practical problems remain unsolved (Sprangers, 2002; Johnson *et al*, 2003). Some of the major issues include item nonresponse and missing data during follow-up. There is not a well-established approach to deal with these problems, but it has been suggested that the use of short instead of lengthy questionnaires may improve the situation (Bernhard *et al*, 1998; Axelsson and Sjoden, 1999; Higginson and Carr, 2001). In a randomised experiment comparing two quality of life questionnaires in a postal survey of stroke patients, patients allocated to the short instrument were significantly more likely to respond and to provide complete data (Dorman *et al*, 1997). In a systematic review of 38 randomised controlled trials, it was found that the use of longer questionnaires was associated with a lower response rate to follow-up by mails (Edwards *et al*, 2004). Our study has demonstrated that it is possible to abridge a quality of life instrument considerably without affecting its measurement properties. Although further research is required to assess whether the use of this instrument can deliver the speculated advantages, the benefit of placing less burden on the part of patients is itself inherently important.

## Figures and Tables

**Table 1:** Patient characteristics

**Characteristics**	**Category/statistics**	**English (*n*=327)**	**Chinese (*n*=221)**
Age	Mean (s.d.)	48.4 (10.1)	50.9 (9.2)
Sex	Female	61.2%	61.5%
Education	Primary or below	1.5%	33.5%
	Secondary	53.8%	49.3%
	Postsecondary	44.7%	17.2%
Tumour site	Breast	37.9%	40.3%
	Lung	8.6%	7.7%
	Colorectal	13.8%	12.7%
	Gynaecological	4.6%	6.3%
	Head and neck (inc. NPC)	17.4%	19.0%
	Others	17.7%	14.0%
ECOG performance score	0	55.1%	28.1%
	1	29.1%	35.8%
	2	13.2%	26.7%
	3 or 4	2.8%	9.5%
On chemo/radiotherapy	Yes	40.1%	34.8%

**Table 2:** Descriptive summary of quality of life scores

	**English**			**Chinese**		
	***N***	**Mean**	**s.d.**	***N***	**Mean**	**s.d.**
Quick-FLIC	327	76.5	17.2	221	76.0	18.8
FLIC	327	76.2	16.4	221	74.8	18.0
FACT-G	167	78.8	13.8	94	75.1	15.5
EORTC	158	67.9	20.6	127	67.3	23.2

FLIC=Functional Living Index – Cancer; FACT-G=Functional Assessment of Cancer Therapy – General; EORTC=European Organisation for Research and Treatment of Cancer.

**Table 3:** Mean (s.d.) values of Quick-FLIC and FLIC (Functional Living Index – Cancer) scores by treatment status and education

	**English**				**Chinese**			
	**Receiving chemo/radiotherapy**		**Postsecondary education**		**Receiving chemo/radiotherapy**		**Postsecondary education**	
	**Quick-FLIC**	**FLIC**	**Quick-FLIC**	**FLIC**	**Quick-FLIC**	**FLIC**	**Quick-FLIC**	**FLIC**
No	80.0	79.4	76.0	75.4	79.5	78.3	76.1	74.8
Yes	71.3	71.3	77.2	77.0	69.4	68.3	75.7	74.7
*P*-value	<0.001	<0.001	0.520	0.388	<0.001	<0.001	0.911	0.969

**Table 4:** Changes in Quick-FLIC and FLIC scores in relation to changes in ECOG performance status

		**English**			**Chinese**		
**Variable**	**Category**	***N***	**Quick-FLIC**	**FLIC**	***N***	**Quick-FLIC**	**FLIC**
Change in ECOG	Improved	49	6.40	6.11	33	5.58	5.96
	Same	131	−0.27	−0.03	89	−2.43	−2.46
	Worsened	47	−8.54	−7.10	39	−9.40	−8.61
	*P*-value		<0.001	<0.001		<0.001	<0.001

FLIC=Functional Living Index – Cancer.

**Table 5:** Reliability of the Quick-FLIC and FLIC

	**English**		**Chinese**	
	**Quick-FLIC**	**FLIC**	**Quick-FLIC**	**FLIC**
Cronbach's alpha	0.86	0.93	0.87	0.93
Test–retest reliability (ICC)	0.76	0.78	0.82	0.83
